# Raoultella planticola Pneumonia: A Rare Causative Organism

**DOI:** 10.7759/cureus.47188

**Published:** 2023-10-17

**Authors:** Jing W Goh, Darryl Braganza Menezes, Rahul Mukherjee

**Affiliations:** 1 Respiratory Medicine, Heartlands Hospital, Birmingham, GBR; 2 Infectious Disease, Heartlands Hospital, Birmingham, GBR

**Keywords:** immunocompetent indivisuals, atypical pneumonia, klebsiella planticola, raoultella planticola, r. planticola

## Abstract

We present a case of *Raoultella planticola* (*R. planticola*) infection that resulted in community-acquired pneumonia in an immunocompetent patient with an eight-week history of productive cough. This gram-negative bacterium is typically found in the environment and has the potential to infect humans. *Raoultella planticola* infections in humans have been recorded in several case reports from throughout the world in recent years, usually affecting immunocompromised patients. Although *R. planticola* is sensitive to most antibiotic groups, recent studies have revealed an increase in the infrequent acquisition of resistance genes in *R. planticola*, such as carbapenem resistance, making this pathogen a potential emergent threat. Our patient acquired *R. planticola* pneumonia in the absence of any underlying risk factors, making this the first case in the UK of *R. planticola* causing community-acquired pneumonia in an immunocompetent person.

## Introduction

*Raoultella planticola (R. planticola)* is a gram-negative aerobic rod found as an environmental reservoir in soil, water, and plants [1]. It was initially termed *Klebsiella planticola* before being renamed *R. planticola* in 2001 based on gene sequencing (16S rRNA and rpoB) [2]. The upper respiratory tract and gastrointestinal tract have always been reservoirs in humans, resulting in pancreatitis, bacteremia, cystitis, and pneumonia [1-2]. Despite the fact that these infections are uncommon, there has been an increase in reports of *R. planticola*-related infections in humans in recent years. Nonetheless, it is still uncommon in the United Kingdom.

## Case presentation

A 41-year-old man who was generally healthy and had no medical issues was admitted to a district general hospital after complaining of coughing up yellow sputum and shortness of breath for eight weeks. The patient is a car dealer with a 20-pack-year smoking history. He had no pets at home and no recent travel or contact history. He was not taking any regular medications, including herbal or over-the-counter remedies that could weaken his immune system. He had no prior hospitalizations or infections that necessitated the use of antibiotics. He experienced no weight loss, changes in appetite, night sweats, palpitation, chest discomfort, or hemoptysis.

On admission, he was tachypneic with a respiratory rate of 24 breaths per minute, febrile with a body temperature of 37.7 degrees Celsius, and hypoxic with an oxygen saturation of 88% on room air. On auscultation of the chest, he had bi-basal coarse crepitation. His admission blood tests revealed 23.8 x10^9^/L of white blood cells (WBC) and 161 mg/L of C-reactive protein (CRP), indicating a possible underlying infection. The rest of the blood tests, including electrolytes, liver and renal function tests, and full blood counts, were all normal. His testing for viral respiratory infections and atypical bacteria, pneumonia, was negative.

**Figure 1:**
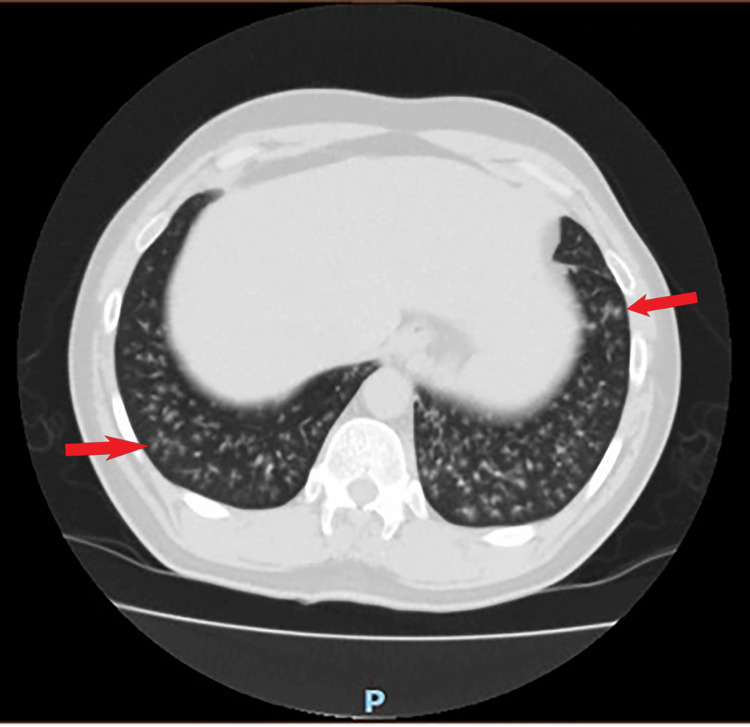
The HRCT shows multiple miliary small nodules in both lung bases. HRCT: high-resolution computed tomography

His chest X-ray revealed nothing unusual. A high-resolution computed tomography (HRCT) two days later revealed multiple nodules with a basal preponderance and minor emphysematous changes in the upper zones (Figure 1).

He was initially diagnosed with a presumed lower respiratory tract infection and treated with intravenous benzylpenicillin and oral clarithromycin

His blood cultures were negative, as were his pneumococcal and legionella urinary antigens. He had four sputum cultures that only grew mixed normal flora and were negative for bacteria and acid-fast bacilli. He was later discharged with oral amoxicillin, clarithromycin, and prednisolone 40mg for seven days and followed up in the respiratory clinic. The HRCT showed multiple miliary small nodules in both lung bases.

Treatment and outcome

In view of the inconclusiveness of the sputum test, bronchial alveolar lavage was performed, which revealed significant growth of *R. planticola* in the bronchial lavage samples. Except for amoxicillin, *R. planticola* was shown to be susceptible to penicillin. It was also sensitive to cephalosporins, aminoglycosides, fluoroquinolones, tetracyclines, and carbapenems. His WBC count decreased from 23.8x10^9^/L to 10x10^9^/L after six days in the hospital while receiving antibiotics. Additionally, CRP improved from 161 mg/L to 6 mg/L. He had a follow-up consultation with a respiratory clinic six weeks after his discharge. He subsequently had a repeat chest X-ray, which showed that his defused parenchymal abnormalities had improved.

## Discussion

*Raoultella planticola* is a rare human pathogen, but it has the potential to be an opportunistic organism, putting immunocompromised individuals at the greatest risk of infection. It has been observed in infections in individuals with diabetes, solid organ transplants, underlying cancer, brain hemorrhage, and pulmonary illness [3-4]. Other than immunosuppression, several risk factors for the disease have been identified, including seafood consumption and invasive medical procedures. Peurta-Fernandez et al. (2012) reported a case of *R. planticola* gastroenteritis that led to bacteremia after consuming poorly prepared seafood [5]. In 2014, Lam et al. reported a similar instance in which *R. planticola* caused bacteriemia after consuming seafood in an immunocompromised patient with lung cancer who was undergoing chemotherapy [6].

Between 2011 and 2017, Hong et al. reported 11 cases of *R. planticola*-caused pneumonia in South Korea, with the majority of the individuals suffering from cancer, chronic obstructive pulmonary disease (COPD), or cerebral infarction/hemorrhage. Consolidation, ground glass opacity, pleural effusion, and micronodules were the most common radiographic abnormalities in these individuals, in that order. Except for one case, all of these individuals recovered after receiving treatment with cephalosporins, carbapenems, fluoroquinolones, aminoglycosides, or beta-lactam/beta-lactamase inhibitors [4]. Interestingly, our patient was immunocompetent and had no medical conditions that would have put him at risk for *R. planticola* infections. He had no recent travel history and had never been exposed to soil, aquatic plants, or seafood. His chest radiograph, on the other hand, revealed numerous micronodules, which is consistent with the radiological changes observed for *R. planticola*.

Antibiotics are frequently chosen based on sensitivity assessments in the treatment of *R. planticola* infections. It has been reported that it is susceptible to a wide range of antibiotics, including cephalosporins, carbapenems, fluoroquinolones, aminoglycosides, and beta-lactams, and hence empirical antibiotics could be used to treat *R. planticola* infection in clinical practice [4]. However, this bacterium has recently shown sporadic multidrug resistance mutations, including carbapenem resistance. Tseng et al. (2014) reported two cases of carbapenem-resistant *R. planticola* that was still susceptible to fluoroquinolone, aminoglycoside, and colistin [7]. In addition, Extended Spectrum Beta-Lactamase (ESBL) *R. planticola* was identified in a recent study [8]. Nonetheless, the majority of the patients are still responsive to the majority of antibiotic groups.

In view of the atypical presentation, propensity for resistance mutations, and potential for disregarding this organism as a colonizer, it is crucial to emphasize the pathogenicity in this case. Therefore, it is essential to raise clinician awareness of *R. planticola*-related infections, which can be fatal for individuals with immunodeficiency.

## Conclusions

*Raoultella planticola* is an uncommon human pathogen that has been reported in increasing numbers in recent studies. Its atypical presentation and proclivity to acquire resistance mutations have made this an important instance to highlight pathogenicity. Although it is susceptible to most antibiotic groups, an emerging carbapenemase-producing *R. planticola* has been identified in recent years, making this pathogen a potential threat to immunocompromised individuals. To the best of our knowledge, this is the first case report of *R. planticola* causing community-acquired pneumonia in a healthy individual in the United Kingdom. Such findings would aid in increasing clinician awareness of the infectious nature of *R. planticola* infection and the need to not disregard it as a colonizer. A case report meta-analysis might assist clinicians in understanding *R. planticola* and the medical disorders it contributes to.
